# Nanofibrous scaffolds for regenerative endodontics treatment

**DOI:** 10.3389/fbioe.2022.1078453

**Published:** 2022-12-12

**Authors:** Fangting Huang, Lei Cheng, Jiyao Li, Biao Ren

**Affiliations:** ^1^ State Key Laboratory of Oral Diseases & National Clinical Research Center for Oral Diseases, Sichuan University, Chengdu, Sichuan, China; ^2^ Department of Preventive Dentistry, Guanghua School of Stomatology, Sun Yat-sen University, Guangzhou, Guangdong, China; ^3^ Department of Operative Dentistry and Endodontics, West China School of Stomatology, Sichuan University, Chengdu, Sichuan, China

**Keywords:** regenerative endodontics treatment, RET, nanofiber, scaffolds, nanomateials

## Abstract

Untreated dental caries, tooth trauma and dental anatomical variations such as dens invaginatus can result in pulpitis. However, standard root canal therapy cannot treat immature permanent teeth due to an open apical foramen and thin dentinal walls. Thus, regenerative endodontics treatment (RET) following a disinfection step with pulp regeneration has been developed. Pulp connective-tissue, dentin formation, revascularization and reinnervation can occur in this procedure which should be supplemented with intelligent biomaterials to improve repeatability and support well-coordinated regeneration. Furthermore, nanofibrous scaffolds, as one of the most commonly used materials, show promise. The purpose of this article is to highlight the advantages of nanofibrous scaffolds and discuss the future modification and application of them.

## 1 Introduction

**FIGURE 1:**
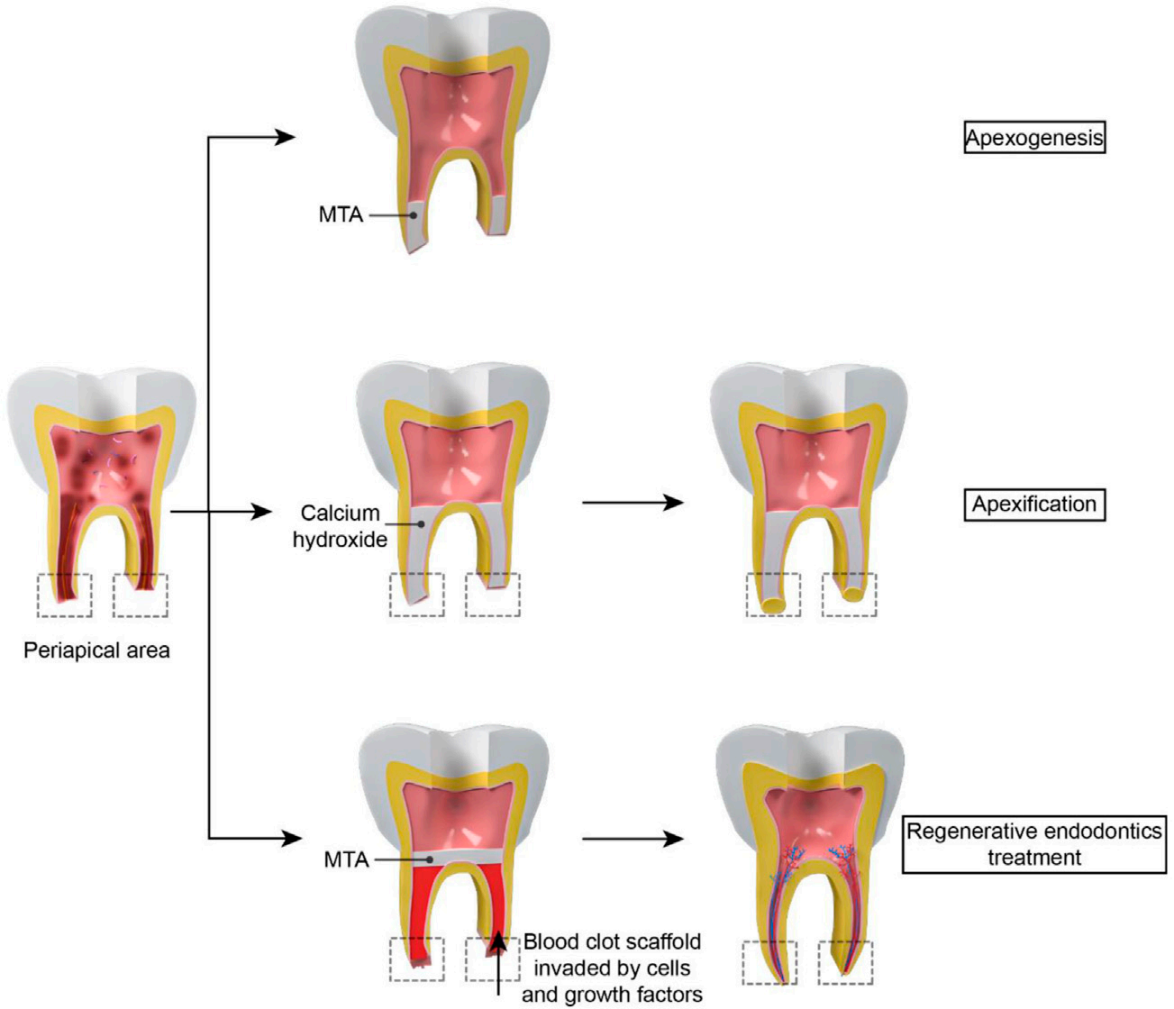
The difference between apexogenesis, apexification and RET: For apexogenesis, MTA is placed in the apical 1/3 of the root canal to form an unnatural barrier. For apexification, biomaterials such as calcium hydroxide is put in the root to stimulate the creation of a mineralized barrier in the root apex. However, vessels cannot regenerate in the pulp cavity. For RET, periapical tissue tearing results in a blood clot which acts as a natural scaffold for stem cells and growth factor to grow into. Then the closure of the apical foramen and/or the thickening of the root canal walls can be observed in the treatment.

Untreated dental caries, tooth trauma, and dental anatomical variations such as dens invaginatus can result in endodontic infections and inflammatory pulpal reactions, a highly prevalent inflammatory condition worldwide (Bletsa et al., 2006; Agnihotry et al., 2016; Estefan et al., 2016). This kind of inflammation can be reversed with appropriate treatment at an early stage (Bjørndal et al., 2014). However, as inflammation progresses, irreversible pulpitis, which manifests as violently acute pain in the tooth, occurs frequently (Lin et al., 2020). If the irreversible pulpits are not treated promptly, it can induce the dental necrosis, apical periodontitis, abscess, and eventual tooth loss (Bjørndal et al., 2019). Current endodontic treatment concepts for fully developed necrotic permanent teeth include removing inflammatory or necrotic pulp tissue and replacing it with biomaterials (Jung et al., 2019). Nowadays, the most popular therapeutic strategy is root canal therapy (RCT), which includes debridement, instrumentation, and obturation of the pulp canal space (Ma et al., 2016; Manfredi et al., 2016). However, the traditional RCT is not recommended for the immature permanent teeth in children due to their thinner dentinal walls and unclosed apical foramen compared to mature permanent teeth (Mc et al., 2019). The apexogenesis/apexification (Figure 1) is an alternative method to solve this challenge. It entails removing all necrotic pulp tissues and utilizing biomaterials such as mineral trioxide aggregate (MTA) or calcium hydroxide (Ca(OH)2) to create an artificial barrier or stimulate the creation of a mineralized barrier in the root apex after enough debridement (Frank, 1966; Heithersay, 1975; Rafter, 2005). However, apexogenesis does not restore the pulp’s vitality and immunocompetence and cannot promote the root maturation (the closure of the apical foramen as mature permanent teeth and/or the thickening of the root canal walls), which may compromise tooth and root wall stability and eventually increase the likelihood of dental secondary trauma, even tooth loss (Andreasen et al., 2002; Kim et al., 2018; Mc et al., 2019). Likewise, apexification cannot ensure the root maturation completely and it need repeated return visits over a long period to determine the final effect for every patient (Rafter, 2005).

Pulp regeneration (Figure 1) is another approach for immature permanent teeth necrosis. A healthy and alive pulp is required for long-term tooth survival and preservation (Schmalz et al., 2020). In 2001, Iwaya et al. (Iwaya et al., 2001) established a novel treatment technique to address a young permanent tooth which was immature and suffered from apical periodontitis and then named it as “revascularization”. In 2007, “regenerative endodontics”, which is based on a tissue engineering notion, was coined by the American Association of Endodontists, indicating this kind of regeneration included both soft and hard tissues rather than vessels (Murray et al., 2007).

Regenerative endodontics treatment (RET) refers to biologically based procedures to replace damaged tooth tissues with newborn tissues (Schmalz et al., 2020). Following a disinfecting phase, RET induces bleeding by periapical tissue tearing. Blood clot can act as a natural scaffold which provide a platform for stem cells to grow into. Various endodontic studies have shown that RET could improve root maturation and reinforce thin and weak young roots (Kahler and Rossi-Fedele, 2016; Chan et al., 2017). RET also led to comparable survival and success rates compared to apexification (Ribeiro et al., 2020). However, this treatment needs the supplement of innovative biomaterials to improve repeatability and encourage a well-orchestrated regeneration (Keller et al., 2015).

RET includes three crucial parts in regeneration engineering: stem cells, bioactive molecules, and scaffolds. Stem cells can serve as a source to differentiate into different cells and form new regeneration-related tissues. Bioactive molecules can promote the cell migration, growth. The scaffold is considered an essential factor in the RET to coordinate drug delivery of active biomolecules or antibiotics and offers a surface for cells to communicate, migrate, proliferate, and spatially organize (Nakashima and Akamine, 2005; Galler et al., 2011).

With the development of pulp regeneration, the scaffolds become one of the key factors to improve the RET’s success rate. Here we summarized the research and development of nanofibrous scaffolds in RET to highlight the advantages of nanofibrous and discuss the future modification and application of nanofibrous.

## 2 Nanofibrous scaffolds

An essential process of the RET is placing a scaffold to generate a three-dimensional (3D) microenvironment for supporting cells, resulting in better outcomes of restoring tissue function (Gupte and Ma, 2012). A variety of materials can be utilized to construct scaffolds in regeneration engineering (Galler et al., 2011). Synthetic polymers [such as polymers of glycolic acid (PGA), polymers of lactic acid (PLA)], and biological matrices (such as reconstituted collagen and fibrin) are two principal alternatives (Galler et al., 2011; Zein et al., 2019). A suitable scaffold should closely be similar to the natural extracellular matrix (ECM), which is the physiological microenvironment of the cells (Gupte and Ma, 2012). ECM is the cell-environmental nanofibrous protein network in all tissues and consists of various nano-scale proteins, such as fibronectin, vitronectin and collagen (Kwon et al., 2005). This matrix can serve as structural support for cell adhesion and ingrowth and give cells chemical signals to govern their activity and reinforce a specific phenotypic (Galler et al., 2011).

Recent breakthroughs in nanotechnology have tremendously aided in creating innovative ECM-mimicking materials. Nanofibrous scaffolds which are becoming increasingly common in the RET can improve adhesion, allow cells to manifest more normal 3D morphologies when compared with smooth materials, solid films, and tissue culture polymers (Gupte and Ma, 2012; Mc et al., 2019). A nanofibrous scaffold is characterized by the diameter ranging from 1 nm to 100 nm, similar to the size of collagen (Sheikh et al., 2015). And it has a large surface area inversely linked to diameter, facilitating cellular adhesion and migration. Furthermore, the pores of nanofibers are suitable for the cell adhesion and penetration. Cells grown on very porous nanofibrous meshes have been proved to increase cellular adhesion and multiply (Kwon et al., 2005). Thus, nanofibrous scaffolds become an ideal material for RET due to nano-scale fibre, large surface area, and interconnected porosity similar to the ECM.

Biodegradability is also an important character for tissue regeneration. Most nanofibrous scaffolds are biodegradable and can be chemically destroyed in the biological environment by enzymes such as lysozyme (Sheikh et al., 2015). The degradability of the nanofibers is determined by two significant factors: the chemical composition of the fibres and hydrophilicity. Therefore, by adjusting these two factors, the biodegradation speed of scaffolds can be controlled in line with the rate of tissue regeneration for better regeneration. The biocompatibility of scaffolds’ broken down chemical compounds matters as well. To better pulp regeneration, the material of the nanofibrous scaffold should be taken into consideration.

Acting as the reservoirs for stem cells, antimicrobial, anti-inflammatory molecules, and growth factors is a considerable advantage for nanofibrous scaffolds (Albuquerque et al., 2015; Keller et al., 2015). Thus, by providing physical and chemical stimulation, nanofibrous scaffolds have been shown to drive positive cell interactions, stimulate cell growth, preserve cell phenotypics, assist stem cell development, and activate cell-signaling pathways (Sun et al., 2010; Gupte and Ma, 2012; Li et al., 2020).

Currently, several materials processing techniques, including electrospinning, molecular self-assembly, and thermally induced phase separation, have also been developed for the production of nanofibrous scaffolds (Gupte and Ma, 2012; Albuquerque et al., 2014).

Electrospinning is extensively employed to make natural and synthetic polymer fibres because of its ease of usage and compatibility with practically any dissolvable polymer. A strong electric field is applied to control the deposition of polymer fibres with diameters ranging from nanometers to micrometers. Fibre morphology, pore size, and fibre alignment may be altered by adjusting electrospinning parameters as well (Yang et al., 2005; Gupte and Ma, 2012). However, it is frequently unable to make real nanofibers at 100 nm or less using commonly used biodegradable polymers. In addition to low production efficiency, factors such as air humidity, temperature, and air velocity in the spinning environment are difficult to control which affect the physical properties of nanofibrous scaffold can also be its shortcomings to achieve industrial production (Sill and von Recum, 2008).

Molecular self-assembly is a bottom-up technique that has been utilized to create nanofibres as small as 10 nm by using spontaneous molecular organization *via* weak non-covalent interactions (Dahlin et al., 2011). Molecular self-assembly nanofibers for RET benefit from being built into solutions and producing gels utilized for stem cell encapsulation (Rosa et al., 2013). Besides, the fluid may be administered *via* a minimally invasive technique, resulting in an in-suit nanofibrous structure. However, this method has limitations in controlling pore size/shape inside the hydrogel scaffold (Gupte and Ma, 2012) and poor mechanical properties in general (Zhang et al., 2012).

Thermally-induced phase separation (TIPS) involves drastically quenching a single-phase homogenous polymer solution, inducing separation into a solvent-rich phase and a polymer-rich phase and with fiber diameters between 50 and 500 nm. It is a technique that has been utilized for a long time to make membranes and scaffolds. This method is common to create nanofibrous scaffolds. TIPS can also be used with other approaches to create macro/micropore/channel networks within 3D nanofibrous scaffolds (Gupte and Ma, 2012).

**FIGURE 2:**
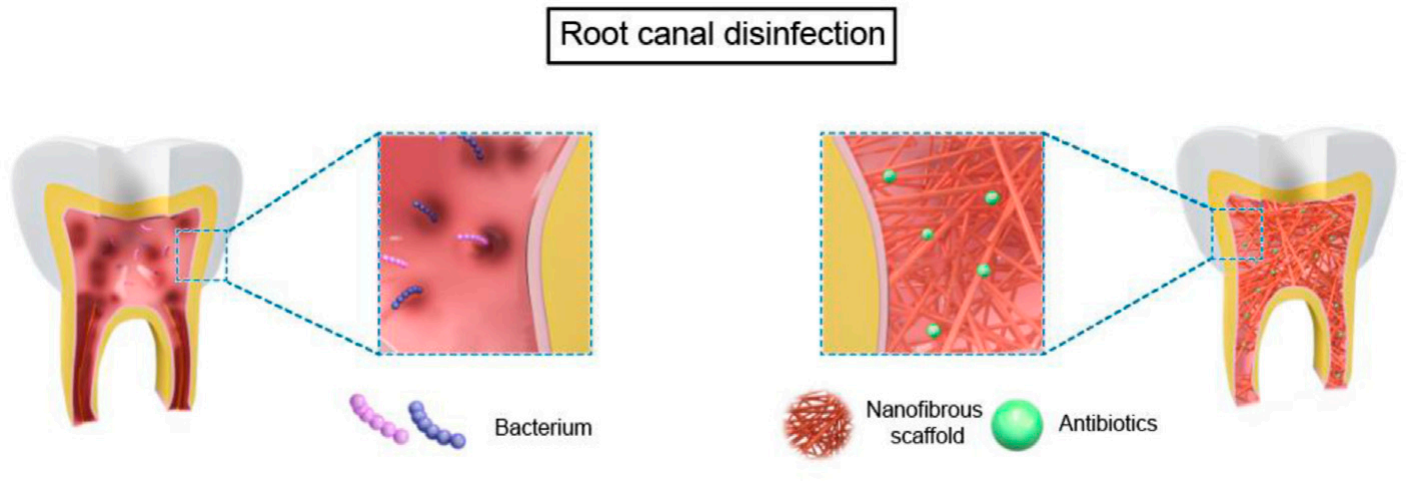
The use of nanofibrous scaffolds in root cannal disinfection.

**FIGURE 3:**
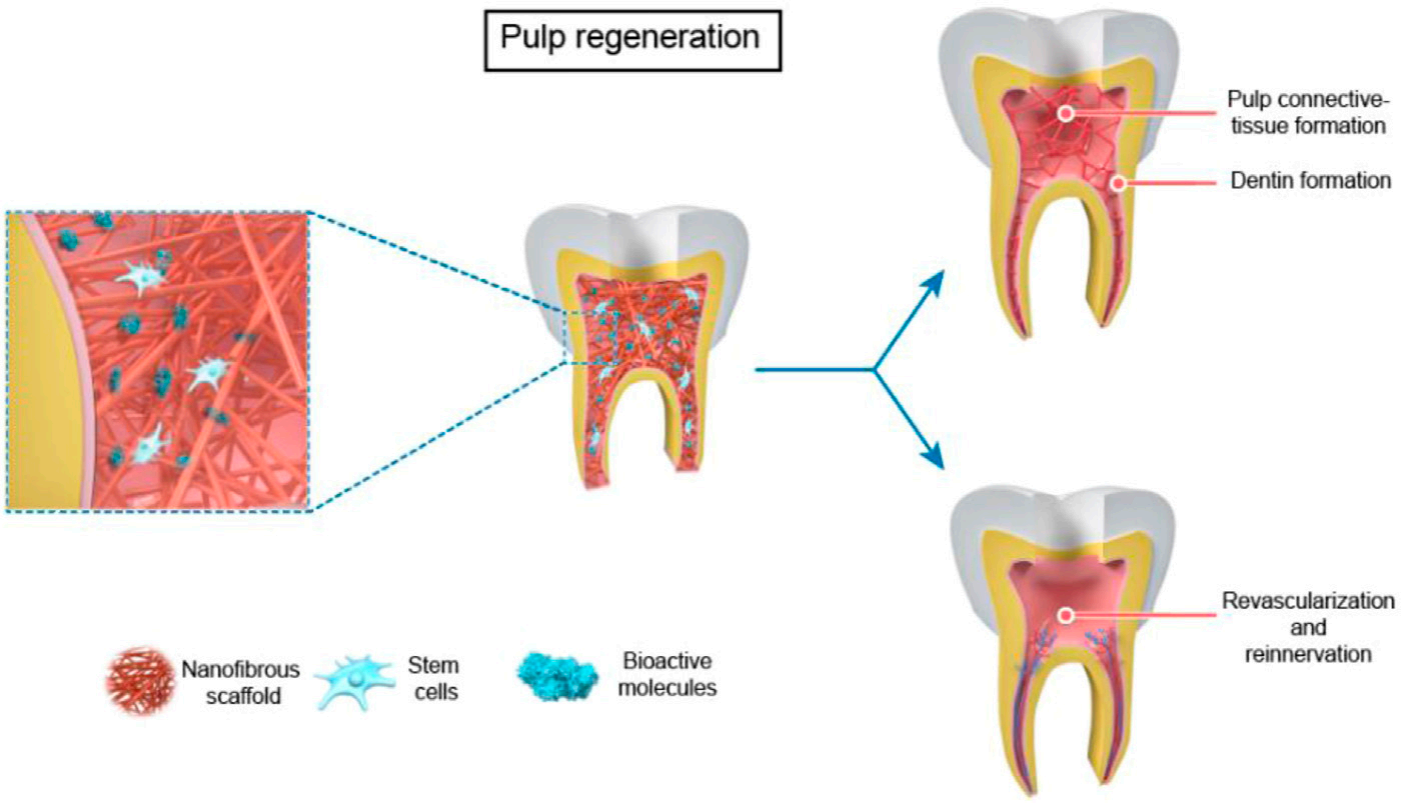
The use of nanofibrous scaffolds in pulp regeneration --- Pulp Connective -Tissue and Dentin Formation, Revascularization and Reinnervation.

The application of nanofiber scaffolds in RET can be devived into two parts in immature teeth based on tissue-engineering-techniques (Gupte and Ma, 2012): 1) bioactive nanofibrous scaffold with antibiotics for root canal disinfection. 2) nanofibrous scaffolds with stem cells and/or biomolecules for dental regeneration in a broad sense, which contains four parts: pulp connective-tissue formation; dentin formation; revascularization; reinnervation (Figure 2, Figure 3).

## 3 Nanofibrous scaffolds for root canal disinfection

Pulp regeneration processes can be quite complex (Schmalz et al., 2020), and bacteria in the root canal further complicates the RET (Kim et al., 2018). Over 600 bacterial species have been identified as potentially involved with pulp infections (Sampaio-Maia et al., 2016). The microbial ecology in the pulp canals of infected young permanent teeth was comparable to that of mature permanen teeth (Nagata et al., 2014). *Enterococcus faecalis* (*E. faecalis*) is commonly isolated from early-root canal infections. *Prophyromonas*, *Treponema*, *Bacteroides*, *Fusobacterium*, *Prevotella*, *Peptostreptococcos*, *Eubacterium*, and *Campylobacter* species are the most commonly found endodontic bacteria in primary endodontic infections (Neelakantan et al., 2017). Bacteria can not only enter the dentinal canal tubules of infected teeth but form biofilms on the radicular canal walls (Lin et al., 2014). Previously, the most commonly used drug in clinical practice for successful disinfection is triple antibiotic paste (TAP), which is made up of equivalent doses of metronidazole (MET), ciprofloxacin (CIP) and minocycline (MINO) (Arruda et al., 2018). The antibacterial effects of this drug are widely recognized in the therapeutic setting, but it has garnered numerous complaints (Kim et al., 2018). One of the most crucial issues reported at TAP was dentin discolouration induced by the presence of MINO (Porter et al., 2016). Therefore, the double antibiotic paste (DAP) combining MET and CIP or the incorporation of ampicillin (AMP) into a new TAP formulation were used (Arslan et al., 2015). However, antibiotic pastes at therapeutically recommended doses will impair the viability of human apical papilla stem cells (SCAPs) (Ruparel et al., 2012). In view of the above disadvantages, Ca (OH)_2_ is commonly used in RET currently. However, it also has several disadvantages. For example, it can form a calcified barrier in the root canal, hindering the growth of pulp tissue (Song et al., 2017; Almutairi et al., 2022). And because of its high pH, it may destroy cells from the apical papilla and periapical tissue as well (Banchs and Trope, 2004; Jadhav et al., 2012).

Thus, it is crucial to employ biomaterials that reduce germs in the root canal while remaining non-cytotoxic and encouraging the creation of new tissues (Murray, 2018). New nano-scaffolds which are capable of serving as drug delivery vehicles in a regulated manner depending on therapeutic use are developed (Sundararaj et al., 2014). Compared to propylene glycol, which is the most often utilized carrier for delivering antimicrobial pastes to the root canal, nanofibers have the potential benefit of increasing the surface area and, presumably, allowing for controllable drug release over a more extended period (Bottino et al., 2013; Palasuk et al., 2014). Figure 2 and Table 1 summarized the use of nanofibrous scaffolds in root canal disinfection.

**TABLE 1:** Nanofibrous scaffolds for root canal disinfection.

Scaffolds	Processing technique	Model	Involved antibiotic	Biocompatiblility		Biodegradability		Results	Reference
				Cells	Cytotoxics	Yes	No		
PSD	electrospinning	*in vitro*: antibacterial experiment, cell cultivation	MET or CIP	hDPSCs	all scaffolds, except 25 wt% CIP group, were considered biologically safe compared with non-antibiotic scaffolds	√		• scaffolds released MET/CIP gradually• electrospinning process did not affect antimicrobial properties• antimicribial activities against *E. faecalis*, *P.gingivalis*	Bottino et al. (2013)
PSD	electrospinning	*in vitro*: antibacterial experiment, cell cultivation	MET and CIP in different proportion	hDPSCs	antibiotic-containing scaffolds had slight toxicity (60%–90% cell viability) or non-cytotoxicity (>90% cell viability)	√		• antimicribial activities against *E. faecalis*, *P.gingivalis*, *F. nucleatum*	Palasuk et al. (2014)
PSD	electrospinning	*in vitro*: cell cultivation	MET or/and CIP in different proportion	hDPSCs	significantly lower effects on cell proliferation and survival compared with the saturated CIP/MET solution	√		• a burst release of antibiotic within the first 24 h, a sustained maintenance of the drug(s) concentration for 14 days• significantly lower effects on cell proliferation and survival compared with the saturated CIP/MET solution	Kamocki et al. (2015)
PSD	electrospinning	*in vitro*	MINO, CIP and MET	hDPSCs	enhanced adhesion/spreading on dentin specimens treated with antibiotic-containing nanofibers when compared with its TAP counterparts	√		• antimicribial activities against *A. naeslundii*, *P.gingivalis* dual-species biofilm whereas not affect *DPSC* attachment and proliferation on dentin	Pankajakshan et al. (2016)
PSD	electrospinning	*in vitro*	MET, CIP and CLIN	hDPSCs	CLIN-containning nanofibers scaffolds showed cell viability above 50%	√		• antimicribial activities against *A. naeslundii, A. actinomycetemcomitans, E. faecalis*• CLIN-containning nanofibers resulted in a slight decrease in hDPSCs viability• no discernibledentin discoloration for the CLIN-containing groups	Karczewski et al. (2018)
PSD	electrospinning	*in vivo* and vitro	MINO, CIP and MET	hDPSCs	histological data showed the scaffolds are biocompatible	√		• antimicribial activities against *A. naeslundii*• apical foramen closure and the formation of a thin layer of osteodentin-like tissue	Mc et al. (2019)

PSD, polydioxanone; MET, Metronidazol; CIP, Ciprofloxacin; hDPSCs, human dental pulp stem cells; CLIN, clindamycin; PCL, poly(e-caprolactone); nHA, nano-hydroxyapatite; MgP, magnesium phosphate; ECM, extracellular matrix; BGNs, bioactive glass nanoparticles; hDPCs, human dental pulp cells; BMP, bone morphogenetic protein; SHED, stem cells from human exfoliated deciduous teeth; hAPCs, human apical papilla cells; PLLA, poly(L-lactic acid); BMP-7, bone morphogenetic protein 7; DXM, dexamethasone; DEX, dexamethasone; PCL-PEG-PCL, poly-caprolactone-poly-ethylene glycol-poly caprolactone; NaF, sodium fluoride; MSH, melanocyte-stimulating hormone; SIM, simvastatin; TGF-β1, transforming growth factor b1; FGF2, fibroblast growth factor basic; VEGF, vascular endothelial growth factor; GFs, the growth factors; HUVECs, human umbilical vein endothelial cells; PLLA, poly(L-lactic acid).

In 2013, Bottino et al. (Bottino et al., 2013) mixed polydioxanone monofilament solution material (PDS-Ⅱ) with MET or CIP in different concentrations (5% or 25%), and typical electrospinning procedures were applied. Almost all scaffolds demonstrated an early burst followed by a general linear continuous drug release. Within the first 48 h, all nanofibrous scaffolds only release a portion of the medication from 22.4% to 51.4% indicated the sustained drug release from the scaffolds. All CIP-containing scaffolds strongly reduced the growth of *Porphyromonas gingivalis* (*P.gingivalis*) and *E. faecalis*. However, MET-containing’s have no *E. faecalis* resistance. Although they all have good antibacterial properties, only the 25% wt CIP-containing extract had a substantial adverse effect on Human dental pulp stem cells (hDPSCs).

A nanofibrous scaffold by combining MET and CIP in different ratios and using PDS as a drug carrier is created in another study (Palasuk et al., 2014). All scaffolds containing antibiotics suppressed the growth of *P. gingivalis*, *E. faecalis*, and *Fusobacterium nucleatum* (*F. nucleatum*), except for the MET-containing scaffold. When compared to scaffolds integrated with individual antimicrobials, scaffolds with two antibiotics did not increase their antimicrobial abilities. Moreover, antibiotic-containing aliquots slightly reduced cell viability to hDPSC.

To further determine the drug release abilities of the new dual-mix antibacterial scaffolds and their effect on the proliferation and activity of hDPSCs. Kamocki et al. (Kamocki et al., 2015) uses electrospinning to create PDS-based nanofibrous scaffolds containing MET and CIP. Pure PDS scaffolds are utilized as the negative (non-toxic) controls, while saturated CIP/MET solution scaffolds are treated as the positive (toxic) groups. The results show no differences in cell proliferation in the pure PDS or MET scaffold groups. For pure CIP-containing scaffolds, increasing drug concentration led to decreased cell viability. However, no cells could be observed after exposing to the saturated CIP/MET solution after 3 days, indicating that antibiotic-containing nanofibrous scaffolds had considerably reduced impacts on hDPSC proliferation. Pankajakshan et al. (Pankajakshan et al., 2016) discovered a similar experimental phenomenon: antibiotic–containing (MINO, CIP, MET) polymer nanofibers resulted in numerous bacterial killing but had no effect on hDPSC adhesion and growth on dentin when compared to a saturated TAP solution. These studies suggest that nanofibrous scaffolds are more biologically friendly to cells when compared with antibiotic paste.

Karczewski et al. (Karczewski et al., 2018) changed the formula of triple antibiotics, including MET, CIP, and clindamycin (CLIN). Hydrated triple antibiotic-containing nanofibrous scaffolds showed pronounced antimicrobial activity against *Actinomyces* naeslundii (A. naeslundii), Aggregatibacter actinomycetemcomitans (A. actinomycetemcomitans), and *E. faecalis*, but with no cytotoxic effects on hDPSC indicating that this kind of triple antibiotic-containing scaffolds might be a feasible option in the RET. Bottino et al. (Mc et al., 2019) optimized electrospinning parameters and created a 3D tubular triple antibiotic-eluting construct to increase the antibacterial activity and the ability to remove bacterial biofilm. The 3D nanofibrous scaffold created an optimal environment with apical foramen closure and a thin layer of osteodentin-like *tissue* within the root canal.

In general, compared with antibiotic pastes, utilizing nanofibrous scaffolds as drug-carrying carriers is more conducive to drug release and increase the capacity of pathogen clearance in the first stage of pulp regeneration. Moreover, it has no toxic effects on stem cells during regeneration. However, there are also some limitations in these researches. The main problem is the lack of *in vivo* studies including antibacterial, degradability and compatibility. Only one article established a canine model of periapical disease. The rest of the studies were still limited to *in vitro* experiments which lack of enough reliability. At the same time, as drug-loaded scaffolds, they also lack the exploration of the best speed for drug release as well as the best degradation rate of the scaffolds which determine the long-term bactericidal ability.

## 4 Nanofibers for dental pulp regeneration in the broad sense

### 4.1 Nanofibrous scaffolds for pulp connective-tissue and dentin formation

Potential cell sources for the RET include hDPSCs, stem cells from human exfoliated deciduous teeth (SHED) and stem cells of the apical papilla (Huang et al., 2008; Morsczeck and Reichert, 2018). These stem cells were incubated with different materials to explore their ability in the RET. Table 2 summarized all the nanofibrous scaffolds used in Pulp Connective-Tissue and Dentin Formation.

**TABLE 2:** Nanofibrous scaffolds for pulp connective-tissue and dentin formation.

Scaffolds	Processing technique	Model	Asscociated tissue engineering strategy		Biocompatibility		Degradability		Results	Reference
			Biomolecules	Cells	Yes	No	Yes	No		
PCL, gelatin and nHA	electrospinning	*in vitro*: cell cultivation *in vivo*: mice subcutaneous transplantation		hDPSCs	√		√		• the scaffolds supported hDPSC adhesion, proliferation, and odontoblastic differentiation• *in vivo* implants were enveloped by a thin fibrous tissue capsule with no negative impacts and increased odontogenic differentiation of stem cells	Yang et al. (2010)
gelatin and silica bioactive glass	thermally induced phase separation	*in vitro*: cell cultivation		hDPSCs	√		√		• compared to nanofibrous gelatin scaffolds, the scaffolds promoted greater cell differentiation and biomineralization	Qu and Liu, (2013)
Gelatin	thermally induced phase separation	*in vitro*: cell cultivation *in vivo*: mice subcutaneous transplantation	MgP	hDPSCs	√		√		• the nanofibrous gelatin/MgP8 above scaffolds produced greater ECM^9^ deposition, hard tissue formation, and odontogenic differentiation protein compared to the nanofibrousgelatin scaffolds	Qu et al. (2014)
PCL	Electrospinning	*in vitro*: cell cultivation		hDPSCs^4^	√		√		• cell growth, mineralization and odontoblastic differetiation on the scaffolds’ surface• the mineralized scaffolds promoting growth and odontogenic differentiation through the integrin-mediated signaling pathway	Kim et al. (2014)
PCL, gelatin, BGNs	electrospinning	*in vitro*: cell cultivation		hDPCs	√		—		• the scaffolds promoted odontogenic differentiation through the integrin, BMP, and mitogen-activated protein kinases signaling path-way	Kim et al. (2015)
Collagen	electrospinning	*in vitro*: cell cultivation		hDPCs	√		√		• scaffolds promoted cell migration, viability, proliferation, adhesion and spreading, as well as collagen production and gene expression	Zhang et al. (2019)
Hydrogel	self-assembling	*in vitro*:cell cultivation	b-glycerophosphate, dexamethasone, KH2PO4	SHED and hDPSCs	√		√		• SHED resulted in soft tissue formation• hDPSCs expressed osteoblast marker genes, and deposited minerals	Galler et al. (2008)
PCL	electrospinning	*in vitro*: cell cultivation	fibronectin	hAPCs	√		—		• The fibronectin added to the scaffolds increase the cell viability, migration, adhesion, growth, and gene expression (ITGA5, ITGAV, COL1A1, COL3A1) at a dose dependent manner	Leite et al. (2021)
PLLA	thermally induced phase separation	*in vitro*: cell cultivation *in vivo*: mice subcutaneous transplantation	BMP-7, DXM	hDPSCs	√		√		• both “DXM” group and “BMP-7 + DXM” group induced the hDPSCs to odontoblast-like cells• “BMP-7 + DXM” group presented more ECM and hard tissue formation than “DXM” group	Wang et al. (2010)
BGNs	electrospinning	*in vitro*: cell cultivation	DEX	hDPSCs	√		√		• The release of DEX showed a slow releasing profile, lasting a month• The scaffolds were demonstrated to promote odontogenesis, and the integrins, bone morphogenetic protein, and mTOR signaling pathways are proposed to be the possible mechanisms	Lim et al. (2016)
PCL-PEG-PCL	electrospinning	*in vitro*: cell cultivation	NaF, MSH, and SIM	hDPSCs	√		—		• significant higher adhesive behavior, viability, alizarin red activity, and dentin specific gene expression in MSH - and SIM - treated cells	Zijah et al. (2017b)

PSD, polydioxanone; MET, Metronidazol; CIP, Ciprofloxacin; hDPSCs, human dental pulp stem cells; CLIN, clindamycin; PCL, poly(e-caprolactone); nHA, nano-hydroxyapatite; MgP, magnesium phosphate; ECM, extracellular matrix; BGNs, bioactive glass nanoparticles; hDPCs, human dental pulp cells; BMP, bone morphogenetic protein; SHED, stem cells from human exfoliated deciduous teeth; hAPCs, human apical papilla cells; PLLA, poly(L-lactic acid); BMP-7, bone morphogenetic protein 7; DXM, dexamethasone; DEX, dexamethasone; PCL-PEG-PCL, poly-caprolactone-poly-ethylene glycol-poly caprolactone; NaF, sodium fluoride; MSH, melanocyte-stimulating hormone; SIM, simvastatin; TGF-β1, transforming growth factor b1; FGF2, fibroblast growth factor basic; VEGF, vascular endothelial growth factor; GFs, the growth factors; HUVECs, human umbilical vein endothelial cells; PLLA, poly(L-lactic acid).

Yang et al. (Yang et al., 2010) seeded hDPSCs on electrospun polycaprolactone (PCL) and gelatin scaffolds with or without nano-hydroxyapatite (nHA). The cells showed the growth and mineralization properties on both the scaffolds. Moreover, a thin fibrous tissue capsule enveloped all implants and osteocyte-like cells and osteoblast-like cells orderly arranged at the scaffolds’ surface in mice subcutaneous transplantation model. And, the incorporation of nHA into scaffolds could increase the expression of specific odontogenic genes.

(Qu and Liu, 2013) created 3D nanofibrous gelatin andsilica bioactive glass (NF-gelatin/SBG) hybrid scaffolds. The hDPSCs proliferated significantly faster on NF-gelatin/SBG scaffolds which promoted better cell differentiation and biomineralization compared to NF-gelatin scaffolds. And the same research group found the 3D NF-gelatin andmagnesium phosphate hybrid scaffolds had a similar effect on hDPSCs which was proved in animal experiments as well (Qu et al., 2014).

(Kim et al., 2014) utilized apatite to deal with the scaffolds’ surface and created mineralized PCL nanofibrous scaffolds. They tested their ability to induce odontogenic differentiation of human dental pulp cells (hDPCs) and the situation of cell adhesion, growth on the scaffolds’ suface. Mineralized PCL scaffold showed better capability in cell growth, mineralized nodule formation, and expression of odontoblastic marker genes compared with the pure PCL scaffolds. And these mineralized scaffolds were appealing for regenerating dentin tissue because they promoted odontogenic differentiation and growth of hDPCs *via* the integrin-mediated signaling pathway. And the same group proved that the bioactive glass nanoparticles - biopolymer blend PCL-gelatin composite nano matrix could promote the odontogenic differentiation of HDPCs by activating the BMP, integrin, and mitogen-activated protein kinase signaling pathway (Kim et al., 2015). Moreover, better ability of promoting cell adhesion, growth and odontoblastic differentiation of hDPCs on collagen nanofibrous matrix (Col_NFM) *versus* collagen flat film (Col-FF) were also observed by Zhang’s group (Zhang et al., 2019).

In addition to using different nanofibrous scaffolds, active biomolecules are also combined with scaffolds to enhance the ability of dental pulp regeneration.

Galler et al. (Galler et al., 2008) combined SHED and hDPSCs with peptide-amphiphile (PA) hydrogel scaffolds which could form 3D nanofibrous networks by self-assemble method. After 4 weeks of cultivating stem cells with different osteogenic supplements, SHED and hDPSCs could proliferate and differentiate within the scaffolds. *In vitro* experiment showed that SHED led to collagen production, while hDPSCs could expressed osteoblast marker genes, manifested an osteoblast-like phenotype, and deposited minerals.

Leite et al. (Leite et al., 2021) developed and evaluated fibronectin (FN)-loaded PCL nanofiber scaffolds of PCL on human apical papilla cells (hAPCs). FN has been postulated as a chemotactic agent that promotes cell migration, adhesion, growth, and differentiation (Grigoriou et al., 2017). The FN added to the scaffolds increase the cell viability, migration, adhesion, growth, and gene expression (ITGA5, ITGAV, COL1A1, COL3A1) at a dose dependent manner.

In order to examine the odontogenic development of hDPSCs, Wang et al. (Wang et al., 2010) fabricated highly porous nanofibrous PLLA scaffolds that resembled the structure of collagen type I fibres. Stem cells were sown onto the scaffolds and grown in various conditions to induce odontogenic differentiation. Both the “DXM” medium (medium containing dexamethasone (DXM)) and “BMP-7+DXM” medium [medium containing DXM, ascorbic acid, β-glycerophosphate plus bone morphogenetic protein 7 (BMP-7)] promoted DPSCs to odontoblast-like cells. After ectopic implantation in nude mice, the “BMP-7 + DXM” group showed greater ECM and complicated tissue development.

The dexamethasone (DEX) - releasing nanofiber matrices with bioactive glass nanoparticles (BGNs) scaffolds were considered a promising therapeutic nano matrix for RET by Lim’s group (Lim et al., 2016). These scaffolds were shown to enhance the odontogenic ability of hDPCs. Bone morphogenetic protein, the integrins, and mTOR signaling pathways were involved in this process (Lim et al., 2016). (Zijah et al., 2017a) aimed to assess and compare the effect of poly-caprolactone-poly-ethylene glycol-poly caprolactone (PCL-PEG-PCL) nanofibrous scaffold containing 3 different biofactors, including sodium fluoride (NaF), melanocyte-stimulating hormone (MSH), and simvastatin (SIM). They found that MSH- and SIM-treated cells had greater adhesive behavior, vitality, dentin-specific gene expression and alizarin red activity.

In terms of nanofibrous scaffolds promoting pulp connective-tissue and dentin formation, we can find scaffolds promoted better cell adhesion, growth, differentiation and biomineralization or high expression of odontoblastic marker genes *in vitro* experiments or mice subcutaneous transplantation model. However, we can’t clearly find the closure of the apical foramen and/or the thickening of the root canal walls in animal experiments. Thus, we need to complete more animal experiments to perfect this defect before clinical trials.

### 4.2 Nanofibrous scaffolds for revascularization and reinnervation

In addition to promoting cell growth, differentiate, nanofibrous scaffolds can also promote angiogenesis and reinnervation in pulp regeneration. The vascularization and reinnervation capabilities of a scaffold are crucial for RET. Table 3 summarized the scaffolds used in revascularization and reinnervation.

**TABLE 3:** Nanofibrous scaffolds for revascularization and reinnervation.

Scaffolds	Processing technique	Model	Asscociated tissue engineering strategy		Biocompatibility		Biodegradability		Results	Reference
			Biomolecules	Cells	Yes	No	Yes	No		
Peptide	self-assembling	*in vitro*:cell cultivation *in vivo*: mice subcutaneous transplantation	TGF-β1, FGF2, and VEGF	hDPSCs	√		√		• cell culture with GFs release from scaffolds showed better cell morphology and proliferation rates• *in vivo* experiment showed the development of a vascularized soft connective tissue	Galler et al. (2012)
peptide hydrogel: PuraMatrix™		*in vitro*: cell cultivation *in vivo*: mice subcutaneous transplantation		HUVECs, hDPSCs	√		√		• co-culturing hDPSCs and HUVECs in PuraMatrix promoted HUVEC survival and induced vessel-like structure formation• *in vivo* experiment showed developed early vascular networks	Dissanayaka et al. (2015)
PLLA	self-assembling	*in vitro*: cell cultivation *in vivo*: mice subcutaneous transplantation	VEGF	hDPSCs	—	√			• hDPSC could attach and proliferate on the scaffolds• *in vivo* experiment showed regenerated pulp-like tissue fulfilled the whole apical	Li et al. (2016)
PCL	electrospinning	*in vitro*: cell cultivation		hDPCs, HUVECs	√		√		• increased cell viability and odontogenic differentiation of HDPCs• increased capillary-like tube formation of HUVECs	Yun et al. (2016)
PLLA	phase-separation	*in vitro*: cell cultivation *in vivo*: subcutaneous implantation mouse model	simvastatin	hDPCs, HUVECs	—	—			• *in vivo* experiment showed formed vessel-like structures• simvastatin could up-regulate odontoblastic markers, exert a pro-angiogenic effect on endothelial cells, resulting in enhanced vascularization and mineralized dentin tissue regeneration	Soares et al. (2018)

PSD, polydioxanone; MET, Metronidazol; CIP, Ciprofloxacin; hDPSCs, human dental pulp stem cells; CLIN, clindamycin; PCL, poly(e-caprolactone); nHA, nano-hydroxyapatite; MgP, magnesium phosphate; ECM, extracellular matrix; BGNs, bioactive glass nanoparticles; hDPCs, human dental pulp cells; BMP, bone morphogenetic protein; SHED, stem cells from human exfoliated deciduous teeth; hAPCs, human apical papilla cells; PLLA, poly(L-lactic acid); BMP-7, bone morphogenetic protein 7; DXM, dexamethasone; DEX, dexamethasone; PCL-PEG-PCL, poly-caprolactone-poly-ethylene glycol-poly caprolactone; NaF, sodium fluoride; MSH, melanocyte-stimulating hormone; SIM, simvastatin; TGF-β1, transforming growth factor b1; FGF2, fibroblast growth factor basic; VEGF, vascular endothelial growth factor; GFs, the growth factors; HUVECs, human umbilical vein endothelial cells; PLLA, poly(L-lactic acid).

Dissanayaka et al. (Dissanayaka et al., 2015) utilized the peptide hydrogel PuraMatrix to test its vascularization ability. In 3D PuraMatrix, they enclosed Human umbilical vein endothelial cells (HUVECs), hDPSCs, or cocultures of these 2 cells and found that hDPSCs aided in developing early vascular networks by promoting the migration of HUVECs and boosting vascular endothelial growth factor (VEGF) expression. Both the hDPSC-only and coculture groups showed vascularized pulp-like tissue with patches of osteodentin in mice subcutaneous transplantation model. Also, Galler et al. (Galler et al., 2012) encapsulated hDPSCs in a self-assembling hydrogel integrated VEGF, fibroblast growth factor primary and transforming growth factor b1. A vascularized soft connective tissue compared to the tooth pulp generated after subcutaneous transplantation within dentin cylinders into mice. Likewise, Li et al. (Li et al., 2016) manufactured a novel hierarchical growth factor-loaded nanofibrous scaffold to solve the vascularization problem. Heparin-binding VEGF was encased in heparin-conjugated gelatin nanospheres, which were subsequently confined in the nanofibers of an injectable PLLA microsphere. This structure shielded the VEGF against degradation and denaturation, and also showed a precise control over its long-term release. After implantation of tooth loaded with nanofibrous scaffolds into immunocompromised nude, the pulp-like tissues filled the whole apical and 2/3 of the root canal, and reached the coronal third. The blood vessels could also be found in the canal.

Yun et al. (Yun et al., 2016) tested the impact of magnetic nanofibrous scaffolds (MNS) on the behaviors of hDPCs. The results indicated that the magnetic scaffolds increased hDPCs development and also promoted the hDPCs-induced angiogenesis of endothelial cells. Soares et al. (Soares et al., 2018) investigated the NF-PLLA scaffolds in mouse model and demonstrated that the addition of simvastatin to a hDPCs/NF-PLLA scaffold might dramatically suppress the production of pro-inflammatory mediators. The detrimental effects of LPS on odontoblastic marker expression might be reversed. Meanwhile, the NF-PLLA nanofibrous scaffolds allowed the HUVECs to form vessel-like structures inside.

For reinnervation, Yoo et al. (Yoo et al., 2018) seeded the hDPCs onto PCLF and PCLF/DMOG dentin slices before transplanting them into mice. The PCLF/DMOG scaffolds increased the expression of VEGF, dentin sialoprotein, bone sialoprotein in the hDPCs, mouse VEGF, mouse neurofilament light polypeptide and mouse platelet endothelial cell adhesion molecule one in the surrounding host cells. These data indicated that pulp-dentin complex regenerated by increasing odontoblastic differentiation of hDPCs, host cell recruitment, neurogenesis and angiogenesis was potentially promoted by PCLF/DMOG.

In conclusion, nanofibrous scaffolds with stem cells and/or active biomolecules promote stem cell-mediated pulp regeneration of pulp connective-tissue formation, dentin formation, revascularization, reinnervation. Currently, nanofibrous scaffolds used for vascular and nerve regeneration are relatively small, and all *in vivo* experiments were still limited to subcutaneous implantation in mice, not in either human or animal pulpless tooth roots. Though, their studies positively showed their regenerative potential to develop early pulp-like tissues by promoting the migration of stem cells and forming vascularized soft connective tissue. However, we still have a long way to go: Does bioparticle-loaded nanofibrous scaffolds have the same effect in immature pulpless teeth? What is the best apical opening size for the scaffolds? What is the optimal degradation rate of the scaffolds for pulp regeneration? In order to avoid cell damage while maintaining the support force of the scaffolds, how much hardness should be selected?

## 5 Conclusion

Currently, there are two significant challenges in the pulp regeneration, including the disinfection of the pulp and the regeneration of tooth-related issues. Regarding pulp disinfection, antibiotic pastes at therapeutically recommended doses impaired the viability of stem cells. And pastes cannot be released at a steady and lasting pace as well. As for the regeneration of tooth-related issues, RET has to be supplemented with scaffolds to improve repeatability and encourage a well-orchestrated regeneration. To address the challenges, nanofibrous scaffolds mimicking extra-cellular matrix (ECM) have been developed. They can act as antibacterial molecule reservoirs and provide growth factors, stem cells to guide the various cells’ migration, growth, and differentiation. However, most of the application of nanofibrous scaffolds is still at the laboratory research stage and lack of the *in vivo* and clinical investigation and evaluation. Meanwhile, we still have a lot of details to work out, including: the optimal degradation rate and drug release rate, the best hardness, suitable physical properties etc. Moreover, no single material can perfectly combine the antibacterial characteristics and stimulate regeneration of connective-tissue, dentin, nerves, and blood vessels simultaneously. New materials are still needed to construct the composite nanofibrous scaffolds for all needs in RET. Furthermore, the *in vivo* and clinical evaluation are also critical for the development of new nanofibrous scaffolds.
